# Impact of the COVID-19 pandemic on medical-seeking behavior in older adults by comparing the presenting complaints of the emergency department visits

**DOI:** 10.1186/s12873-023-00819-5

**Published:** 2023-06-06

**Authors:** Henry Chih-Hung Tai, Yi-Hao Kao, Yen-Wen Lai, Jiann-Hwa Chen, Wei-Lung Chen, Jui-Yuan Chung

**Affiliations:** 1 Department of Emergency Medicine, Cathay General Hospital, Taipei, Taiwan; 2 School of Medicine, Fu Jen Catholic University, Taipei, Taiwan; 3 Department of Emergency Medicine, Sijhih Cathay General Hospital, New Taipei city, Taiwan; 4 School of medicine, National Tsing Hua University, Hsinchu, Taiwan

**Keywords:** Coronavirus disease 2019, Emergency department, Chief complaints

## Abstract

**Background:**

The outbreak of the coronavirus disease 2019 (COVID-19) has caused a catastrophic event worldwide. Since then, people’s way of living has changed in terms of personal behavior, social interaction, and medical-seeking behavior, including change of the emergency department (ED) visiting patterns. The objective of this study was to analyze the impact of the COVID-19 pandemic on the ED visiting patterns of the older people to explore its variable expression with the intention of ameliorating an effective and suitable response to public health emergencies.

**Methods:**

This was a retrospective study conducted in three hospitals of the Cathay Health System in Taiwan. Patients aged ≥ 65 years who presented to the ED between January 21, 2020, and April 30, 2020 (pandemic stage), and between January 21, 2019, and April 30, 2019 (pre-pandemic stage) were enrolled in the study. Basic demographics, including visit characteristics, disposition, and chief complaints of the patients visiting the ED between these two periods of time, were compared and analyzed.

**Results:**

A total of 16,655 older people were included in this study. A 20.91% reduction in ED older adult patient visits was noted during the pandemic period. During the pandemic, there was a decrease in ambulance use among elderly patients visiting the ED, with the proportion decreasing from 16.90 to 16.58%. Chief complaints of fever, upper respiratory infections, psychological and social problems increased, with incidence risk ratios (IRRs) of 1.12, 1.23, 1.25, and 5.2, respectively. Meanwhile, the incidence of both non-life-threatening and life-threatening complaints decreased, with IRRs of 0.72 and 0.83, respectively.

**Conclusion:**

Health education regarding life-threatening symptom signs among older adult patients and avocation of the proper timing to seek medical attention via ambulance were crucial issues during the pandemic.

## Background

In December 2019, coronavirus disease 2019 (COVID-19) spread globally at an unprecedented speed, leading to the beginning of a worldwide pandemic, which had profound and yet still unfolding health and socioeconomic impacts. The impact of the COVID-19 pandemic caused by severe acute respiratory syndrome coronavirus 2 (SARS-CoV-2) virus is rapidly approaching the magnitude of the 1894-plague (12 million deaths) and 1918-A (H1N1) influenza (50 million deaths) pandemics [1].

Since the outbreak of COVID-19 in late December 2019, catastrophic events have spread worldwide amid emotional, economic, and medical tensions. In January 21, 2020, the first imported case of COVID-19 was reported in Taiwan [2]. In February 28, 2020, the first in-hospital COVID-19 cluster infection occurred and the peak pandemic period commenced. The number of patients with COVID-19 rapidly increased from March to April 2020. The number of confirmed COVID-19 cases in Taiwan reached 759 by December 19, 2020 [3].

The COVID-19 pandemic has caused social and economic tension that affects people’s health and well-being. It is widely acknowledged that older adults are at a higher risk of experiencing negative outcomes, including mortality and severe illness, related to the pandemic. This increased vulnerability is attributed to their impaired immune system and the presence of multiple underlying health conditions, also known as multi-comorbidities, when compared to younger individuals [4, 5]. The exponential increase in new cases has overwhelmed healthcare services with extreme safety precautions, leading to a change in the general population’s behavior in seeking medical care. There is only limited evidence of studies that evaluate the medical-seeking behaviors in the older adult population by comparing the presenting complaints of emergency department (ED) visits during times of crisis. These results may benefit in improved medical resource utilization during pandemic situations, especially for older adult patients.

## Methods

### Study setting

A retrospective cross-sectional study was performed by analyzing the medical history database of the Cathay Health System hospitals, including medical centers, regional hospitals, and district hospitals in northern Taiwan. Three hospitals were recruited for the study. One urban medical center is located in Taipei city, with an 800-bed capacity and an estimated total annual ED visit volume of 60,000. The other two included hospitals are both located in rural areas in New Taipei city and Shin-Chu city. Each hospital has a 642- and 348-bed capacity with estimated annual ED visiting volumes of 48,000 and 30,000, respectively [6]. Patients, age ≥ 65 years, visiting the ED of these three hospitals between January 21, 2020, and April 30, 2020, and between January 21, 2019, and April 30, 2019, will be included [6].

### Study design

Patient data were extracted from the electronic medical record (EMR) system during the period between January 21, 2020 and April 30, 2020, as the “pandemic” group, against the similar period 1 year prior to the pandemic as the “pre-pandemic” group. The pandemic period was set up by the first confirmed case in Taiwan on January 21, 2020, and ended on April 30, 2020, which was the 4th day after there were no confirmed cases for 3 consecutive days. The inclusion criteria for this study were as follows: (1) all older adult patients aged 65 years or older, and (2) patients who presented to the emergency department (ED) during the defined “pandemic” and “pre-pandemic” periods. To ensure data integrity, any missing or duplicate data (patients with 72-hours returned ED visit) will be excluded as part of our exclusion criteria.

### Variables

The basic demographics of patients presenting in these two periods were obtained, including visit characteristics, personality, and major complaints. Visiting characteristics consisted of total daily visits, mode of arrival, and time of visit. Chief complaints were based on patients’ narratives recorded on the EMR and sorted into 33 common discomforts [6]. The primary outcome of this study is to compare and identify differences between these two periods in terms of patient demographics, visit characteristics, and chief complaints. Upon arrival at the ED, patients were categorized into five different triage levels, from triage level 1 to triage level 5 according to the Taiwan Triage and Acuity Scale (TTAS) by a qualified triage nurse [6]. The TTAS is a system used to categorize patients into different levels of urgency based on their condition and the severity of their symptoms. This system includes five triage levels, with level 1 being the most urgent (for patients who require immediate resuscitation) and level 5 being the least urgent (for patients who do not require immediate medical attention); while level 2 to level 4 represent for emergent, urgent, and less urgent, respectively. The TTAS is based on the previous Taiwan Triage System (TTS) and has been modified under the framework of the Canadian Triage and Acuity Scale (CTAS) [7]. All triage nurses were trained with the completion of triage fundamentals courses and had more than 1 year of ED working experience.

### Ethical statement

The study was approved by the Institutional Review Board of the Cathay General Hospital Bioethical Committee and was conducted in accordance with the Declaration of Helsinki, the approval number: CGH-P109047. This was an observational study that added no extra intervention to the usual care of patients. Therefore, the requirement for informed consent was waived by Institutional Review Board of the Cathay General Hospital Bioethical Committee.

### Statistical analysis

IBM Statistical Package for the Social Sciences (SPSS) Version 25.0 was used in the analysis. Categorical variables are presented as numbers and percentages, whereas normally distributed continuous variables are presented as mean ± standard deviation. The chi-square test was used to analyze categorical variables, and an independent t-test was used to analyze normally distributed continuous variables. We calculated the incidence rate (number of results of interest divided by the total number of ED visits in each period), incidence rate ratio (IRR), and percentage difference (compares the raw number of cases before and during the pandemic periods) in the number of chief complaints between the “pre-pandemic” period and the “pandemic” periods.

## Results

**Fig. 1:**
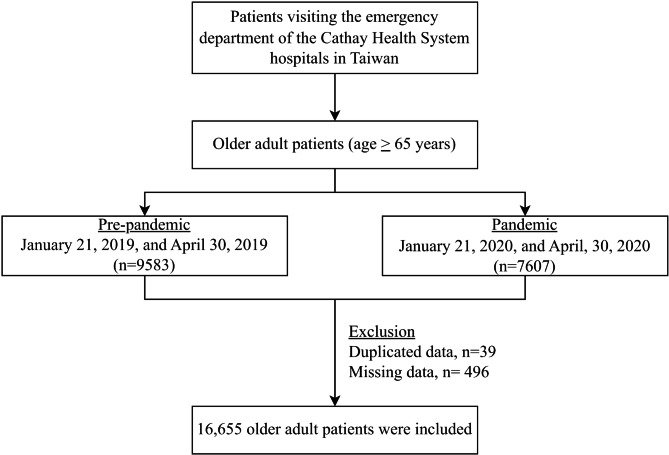
Flowchart of this study

A total of 17,190 older adult patients were included initially, after excluding 39 duplicated and 496 missing cases, 16,655 patients were ultimately included in this study (Fig. 1). There were 9,300 ED visits before the pandemic period between January 21, 2019, and April 30, 2019; a 21.7% (7,355 ED visits) reduction rate in ED visits was noted during the pandemic period (Fig. 1). During the pandemic, the daily ED visits of both male and female older adult patients had decreased prominently from 41.09 ± 8.06 visits per day to 33.46 ± 8.49 visits per day and 51.91 ± 10.20 visits per day to 39.37 ± 10.86 visits per day, respectively. Regarding modes of arrival, the proportion of ambulance usage during the pandemic period decreased slightly from 16.90 to 16.58%. In contrast, the percentage of walk-in patients increased from 53.47 to 60.53%.

The proportion of level 1 and level 5 patients increased from 5.04% and 0.7–5.70% (*p* < 0.01) and 0.98% (*p* = 0.07), respectively, during the pandemic. A decrease in the percentage of level 2 to level 4 patients was also observed with. The mortality rate increased from 66 cases (0.71%) to 83 cases (1.13%) during the pandemic. The majority of older adult ED visits occurred during the day in both the pre-pandemic and pandemic periods. The percentage of ED visits during nighttime decreased from 33.15 to 30.44% during the pandemic compared to the pre-pandemic period (Table 1).

**Table 1:** Comparison of demographic characteristics of emergency department visits by older adult patients between two time periods

Characteristics	Pre-pandemic (Jan 2019–Apr 2019) (N = 9,300)	Pandemic (Jan 2020–Apr 2020) (N = 7,355)	*p*-value
Total visits/day — no. (SD)	93.00 (15.19)	72.82 (17.47)	< 0.01
Sex (daily visits) — no. (SD)			
Male	41.09 (8.06)	33.46 (8.49)	< 0.01
Female	51.91 (10.20)	39.37 (10.86)	< 0.01
Mode of arrival* — no. (%)			
Walk-in	4,973 (53.47)	4,452 (60.53)	< 0.01
Wheelchair	2,520 (27.95)	1,473 (20.74)	< 0.01
Ambulance	1,524 (16.90)	1,178 (16.58)	< 0.01
Triage — no. (%)			
Triage level 1	469 (5.04)	419 (5.70)	< 0.01
Triage level 2	1,842 (19.81)	1,425 (19.37)	< 0.01
Triage level 3	6,314 (67.89)	4,967 (67.53)	< 0.01
Triage level 4	610 (6.56)	472 (6.42)	< 0.01
Triage level 5	65 (0.70)	72 (0.98)	0.07
Disposition — no. (%)			
Admission	2,618 (28.15)	2,084 (28.33)	< 0.01
Discharge	6,286 (67.59)	4,951 (67.31)	< 0.01
AMA	255 (2.74)	174 (2.37)	< 0.01
Transfer	75 (0.81)	63 (0.86)	0.29
Mortality	66 (0.71)	83 (1.13)	0.15
Time of visit — no. (%)			
Early morning (00.00−08.00)	1,328 (14.28)	988 (13.43)	< 0.01
Day time (08.00−17.00)	4,889 (52.57)	4,128 (56.13)	< 0.01
Nighttime (17.00−24.00)	3,083 (33.15)	2,239 (30.44)	< 0.01

* Some records missed the mode of arrival

SD, standard deviation; AMA, against medical advice

Older adult ED-visiting patients with the chief complaints of fever and upper respiratory infection, which mimic the COVID-19 presenting symptom signs, were 1.12 and 1.23 times higher in the pandemic period, respectively, compared to the pre-pandemic period. Gastrointestinal, cardiovascular, and neurological complaints had risk ratios of 0.89, 0.86, and 0.74, respectively. However, the chief complaints of abdominal pain and convulsions increased during the pandemic by 11.24% and 0.44%, respectively, compared with 9.90% and 0.37%, respectively, before the pandemic (Table 2).

**Table 2:** Chief complaints of total emergency department visits by older adult patients between two time periods

Chief Complaints	Pre-pandemic (Jan 2019–Apr 2019) N = 9,300 (Incidence)	Pandemic (Jan 2020–Apr 2020) N = 7,355 (Incidence)	Difference (%)		Incidence rate ratio (%)	*p*-value	
**Infection-related complaints**							
Fever	1,018 (10.95)	905 (12.30)	-11.10	1.12			0.02
URI	685 (7.37)	670 (9.11)	-2.19	1.23			0.72
Cellulitis	204 (2.19)	155 (2.11)	-24.02	0.96			0.01
**Gastrointestinal complaints**							
Abdominal pain	921 (9.90)	827 (11.24)	-10.21	1.14			0.03
AGE symptoms	651 (7.00)	307 (4.17)	-52.84	0.60			< 0.01
Constipation	100 (1.08)	50 (0.68)	-50.00	0.63			< 0.01
GIB symptoms	228 (2.45)	154 (2.09)	-32.46	0.85			< 0.01
**Cardiovascular complaints**							
Chest pain	661 (7.11)	520 (7.07)	-21.33	0.99			< 0.01
Hypertension	273 (2.94)	100 (1.36)	-63.37	0.46			< 0.01
Shortness of breath	900 (9.68)	635 (8.63)	-29.44	0.89			< 0.01
**Neurological complaints**							
Dizziness	975 (10.48)	523 (7.11)	-46.36	0.68			< 0.01
Headache	154 (1.66)	83 (1.13)	-46.10	0.68			< 0.01
Convulsion	34 (0.37)	32 (0.44)	-5.88	1.19			0.77
Stroke symptoms	180 (1.94)	135 (1.84)	-25.00	0.95			0.01
Altered mental status	244 (2.62)	150 (2.04)	-38.52	0.78			< 0.01
Malaise	259 (2.78)	135 (1.84)	-47.88	0.66			< 0.01
Myalgia	534 (5.74)	353 (4.80)	-33.90	0.84			< 0.01
Glycemic problems	131 (1.41)	65 (0.88)	-50.38	0.62			< 0.01
Urological symptoms	372 (4.00)	238 (3.24)	-36.02	0.81			< 0.01
Medical device problems (tube, probe, or catheter)	457 (4.91)	260 (3.54)	-43.11	0.72			< 0.01
Trauma	1,534 (16.49)	1,544 (20.99)	0.65	1.27			0.93
Facial feature problems*	157 (1.69)	133 (1.81)	-15.29	1.07			0.21
OBS-GYN related problems	7 (0.08)	34 (0.46)	385.71	5.75			< 0.01
Dermatology problems	137 (1.47)	115 (1.56)	-16.06	1.06			0.23
Cardiac arrest	76 (0.82)	56 (0.76)	-2.19	0.93			0.06
Transfer from OPD and LMD	364 (3.91)	341 (4.64)	-6.32	1.19			0.48
Transfer from hospital	144 (1.55)	91 (1.24)	-36.81	0.80			< 0.01
Psychological problems	37 (0.40)	37 (0.50)	0.00	1.25			0.97
Social problems†	5 (0.05)	19 (0.26)	280.00	5.2			< 0.01
Others	79 (0.85)	53 (0.72)	-32.91	0.85			0.05

* Facial feature problems, including eyes, ears, nose, and throat (ENT), and dental problems

† Social problems, including family violence and sexual assault

URI, upper respiratory infection; AGE, acute gastroenteritis; GIB, gastrointestinal bleeding; OBS-GYN, obstetrics-gynecology; OPD, outpatient department; LMD, local medical department

Glycemic, urological, and medical device problems significantly decreased during the pandemic period from 1.41%, 4.00%, and 4.91–0.88%, 3.24%, and 3.54%, respectively. The IRR of trauma, facial feature problems, dermatological problems, and psychological problems visits were 1.27, 1.07, 1.06, and 1.25, respectively, without statistical significance. The percentage of patients visiting the ED for obstetrics-gynecology and social problems increased significantly during the pandemic from 0.08% to 0.05% to 0.46 and 0.26, respectively, with IRR of 5.75 and 5.2, respectively (Table 2).

## Discussion

Several studies have shown a significant reduction in ED visits related to different disciplines during the first few weeks of the pandemic worldwide [8]. The general opinion is that patients avoid hospitals for the fear of contracting SARS-CoV-2. In particular, older adults with weakened immune systems are at a higher risk of complications and severe illness if they contract the virus [9]. In our study, we found that during the pandemic, the total number of older adult patients with ED visits at all Cathay Health System hospitals decreased substantially. Several factors, including social distancing policies, travel and gathering restrictions, business and school closures, quarantine measures, and media influence, have contributed to the decline in total ED visits.

According to our research, during the pandemic, a decreased proportion of ambulance use was observed in older adult patients visiting the ED. Although some studies showed a decreased proportion of older adults arriving at the ED by ambulance [10], a similar result was noted in a retrospective study from Denmark, which revealed a 10.3% reduction in ambulance call volume [11]. Another study from Italy also showed a reduction in the number of emergency ambulance services during the pandemic [10]. These results may be due to insufficient ambulance services and fear of being infected in the ambulance during transport to the ED. To prevent delayed medical attention due to the fear of infection, proper education about COVID-19 should be publicized by either social media or cellphone messages, and the accurate timing of seeking medical attention via ambulance should also be advocated.

In the current study, the proportion of level 1 older adult patients visiting ED, despite not significant, and the mortality rate increased during the pandemic compared to those in the pre-pandemic period. This might be attributed to the lockdown policy and fear of infection, which discouraged people from seeking medical attention, especially older adult patients with disabilities. Delayed ED visits until the patient’s condition becomes critical may result in a high acuity level of visit and death rate [12].

Non-life-threatening complaints, such as glycemic problems, urological problems, and medical device problems, have reduced the proportion of ED visits during the pandemic, probably due to the fear of contracting COVID-19 in medical facilities, especially in crowded ED [13]. However, the fear of the unknown virus with constant media reports may, in contrast, drive the patients to visit the ED for the chief complaints that are correlated with COVID-19, including fever and upper respiratory infection [14].

Life-threatening complaints, such as cardiovascular and neurological complaints, showed a slight decrease in the present study during the pandemic period, which may be related to the fear of contagion, which dissuades patients from seeking medical attention [12]. Acute conditions, such as trauma, slightly increased the IRR during the pandemic without significance in this study. Most other studies showed a reduction in older adult trauma cases before and during the pandemic due to the stay-at-home policy and restricted mobility during the pandemic [15, 16]. Health education regarding life-threatening symptom signs among the older adult patients should be provided to caregivers to inform them to seek timely medical attention.

Chief complaints related to psychological and social problems increased during the pandemic. Social problems, including mistreatment (physical abuse, neglect, and sexual abuse), are important issues for older adult population, which may result in ED visits. The frequency and severity of mistreatment in older adult population have increased during the pandemic. This might be attributed to the stay-at-home orders or quarantine period that trapped older people at home with abusers. Furthermore, unemployment, decreased income, and increased stress for family caregivers were very common during the pandemic [17]. Policies should be established to prevent such social problems, especially mistreatment of older adult population during the pandemic.

This study has some limitations. First, some important data were not available because of the retrospective design of this study. Second, formal triage training is mandatory for all triage nurses and individual biases may occur among different triage nurses when triaging patients. Third, this study was conducted in three different hospitals in northern Taiwan and may not be generalizable to other regions. Therefore, further studies are needed to validate our results.

## Conclusions

During the pandemic, three important actions should be performed to prevent delayed medical attention in the vulnerable older adult population due to the fear of infection. First, proper education about COVID-19 should be publicized by social media. Second, health education on the life-threatening symptom signs among the older adult patients should be provided. Third, the accurate timing to seek medical attention via ambulance should be advocated, especially in older adult population. Furthermore, the government should establish policies to prevent social problems, especially mistreatment of older adult population, during the pandemic.

## Data Availability

The datasets used and/or analysed regarding to the current study are available from the corresponding author on reasonable request.

## Abbreviations

COVID-19: Coronavirus disease 2019
ED: Emergency department
EMR: Electronic medical record
EMS: Emergency medical service
IRR: Incidence rate ratio
